# The relationship between sleep quality, snoring symptoms, night shift and risk of stroke in Chinese over 40 years old

**DOI:** 10.3389/fnagi.2023.1134187

**Published:** 2023-04-04

**Authors:** Yajing Zhang, Ting Zhang, Xiaoshuang Xia, Yahui Hu, Chao Zhang, Ran Liu, Yun Yang, Xin Li, Wei Yue

**Affiliations:** ^1^Department of Neurology, Tianjin Huanhu Hospital, Tianjin, China; ^2^Department of Neurology, The Second Hospital of Tianjin Medical University, Tianjin, China

**Keywords:** sleep quality, snoring symptoms, night shift, stroke, old

## Abstract

**Objectives:**

To analyze the relationship between sleep quality, snoring symptoms, night shift and risk of stroke in Chinese population over 40 years old.

**Methods:**

Based on the national screening and intervention program for high-risk population of stroke in 2016, 15,016 people completed the study of “the association between sleep and stroke,” 58,696 people completed the snoring questionnaire, and 58,637 people completed the night shift questionnaire.

**Results:**

The proportion of coronary heart disease, hypertension, hyperlipidemia, diabetes, snoring, atrial fibrillation, stroke and high-risk group of stroke risk rating were higher in the group with poor sleep quality (*p* < 0.05). The proportion of high blood pressure, hyperlipidemia, diabetes, atrial fibrillation, transient ischemic attack (TIA), or high-risk group of stroke risk rating was higher in snoring group (*p* < 0.05). The body mass index (BMI), waist circumference, neck circumference, fasting blood glucose, triglyceride (TG), total cholesterol (TC), low density lipoprotein (LDL) and homocysteine (Hcy) levels in snoring group were higher than the non-snoring group, and high density lipoprotein (HDL) levels were lower (*p* < 0.05). People with TIA, high risk for stroke, and high blood pressure were higher in night shift workers than non-night shift workers (*p* < 0.05). The levels of BMI, fasting blood glucose, 2 h postprandial blood glucose, glycated hemoglobin, TG, TC, LDL, HDL and Hcy in night shift group were lower than the non-night shift group (*p* < 0.05).

**Conclusion:**

Sleep quality, snoring and night shift might be related to the risk factors of stroke.

## Introduction

One third of human life is spent in sleep and sleep is important for everyone. Up to 150 million people in the word suffer from sleep problems that can affect their quality of life and make them vulnerable to other adverse consequences (Lawes et al., 2008). Stroke is a common cerebrovascular disease. There were about 16 million people in the world experienced their first stroke every year, among which about 5.7 million people died and about 5 million people were left with disabilities.

Sleep quality is associated with a variety of factors, including diabetes, high blood pressure, stroke, myocardial infarction, chronic kidney disease, depression, cognitive impairment, pain, and lifestyle habits such as alcohol use, smoking, and certain physical activities. Sleep quality is associated with stroke, hypertension and so on. In a meta-analysis of 16 prospective studies, a “J” shaped trend was observed between total sleep time and stroke and the lowest risk of stroke was found in people who slept for 7 h at night (He et al., 2017). A prospective study of 1,268 patients with hypertension in 2010 found that the risk of stroke in hypertensive patients with total sleep time <7.5 h is two times higher than that in hypertensive patients with total sleep time ≥7.5 h (Eguchi et al., 2010).

Snoring is a form of sleep-disordered breathing, and snoring can lead to decreased sleep quality. Snoring may have adverse effects on healthy. One study showed that habitual snorers had a 26% increased risk of stroke and a 15% increased risk of coronary heart disease (Li et al., 2014). In a case-control study of 400 patients hospitalized for stroke, snorers are 3.2 times more likely to have a stroke than non-snorers (Spriggs et al., 1992). In a case-control study of 177 stroke patients and 177 age—and sex-matched patients, habitual snoring was found to be an independent risk factor for stroke with an OR of 2.1 (Palomäki, 1991).

Night shift can disrupt circadian rhythm, impair sleep quality, affect the balance between work and life, and is closely related to the occurrence of coronary heart disease and stroke. A report from the Nurses' Health Study showed that night nurses had a 4% increased risk of ischemic stroke (Brown et al., 2009). A prospective cohort study of ~500,000 Finnish men showed that night work was associated with an increased risk of death from cerebrovascular disease compared with normal day work (Virtanen and Notkola, 2002). Another study showed that the relative risk of cardiovascular disease among night shift workers was 1.4 times that of non-night shift workers (Bøggild and Knutsson, 1999). Night shift workers have a higher incidence of hypertension and a higher incidence of high triglyceride (Ruidavets et al., 1998).

Previous studies have linked sleep quality, snoring symptoms and night shifts to stroke, coronary heart disease, hypertension and metabolic syndrome. The purpose of this study is to investigate the sleep quality, snoring symptoms and night shift of people over 40 years old in Chinese community, to analyze the risk factors of stroke among people with poor sleep quality, snoring symptoms and night shift, and to explore the relationship between sleep quality, snoring symptoms, night shift and risk of stroke. May be in the future, the diagnosis and treatment of sleeping disorders should be considered as a prevention and intervention of stroke.

## Subjects and methods

All data were from the National Major Public Health Service Project-2016 Annual Screening and Intervention Program for Stroke Patients at High Risk (CN-2016F0007). The data were extracted from the database platform of “Chinese Stroke Center,” and the use consent of the data center was obtained. A total of 31 provinces, autonomous regions, municipalities and Xinjiang Production and Construction Corps were included and a total of 423,603 community residents over 40 years of age completed the program screening from April 2016 to May 2017.

**Figure 1:**
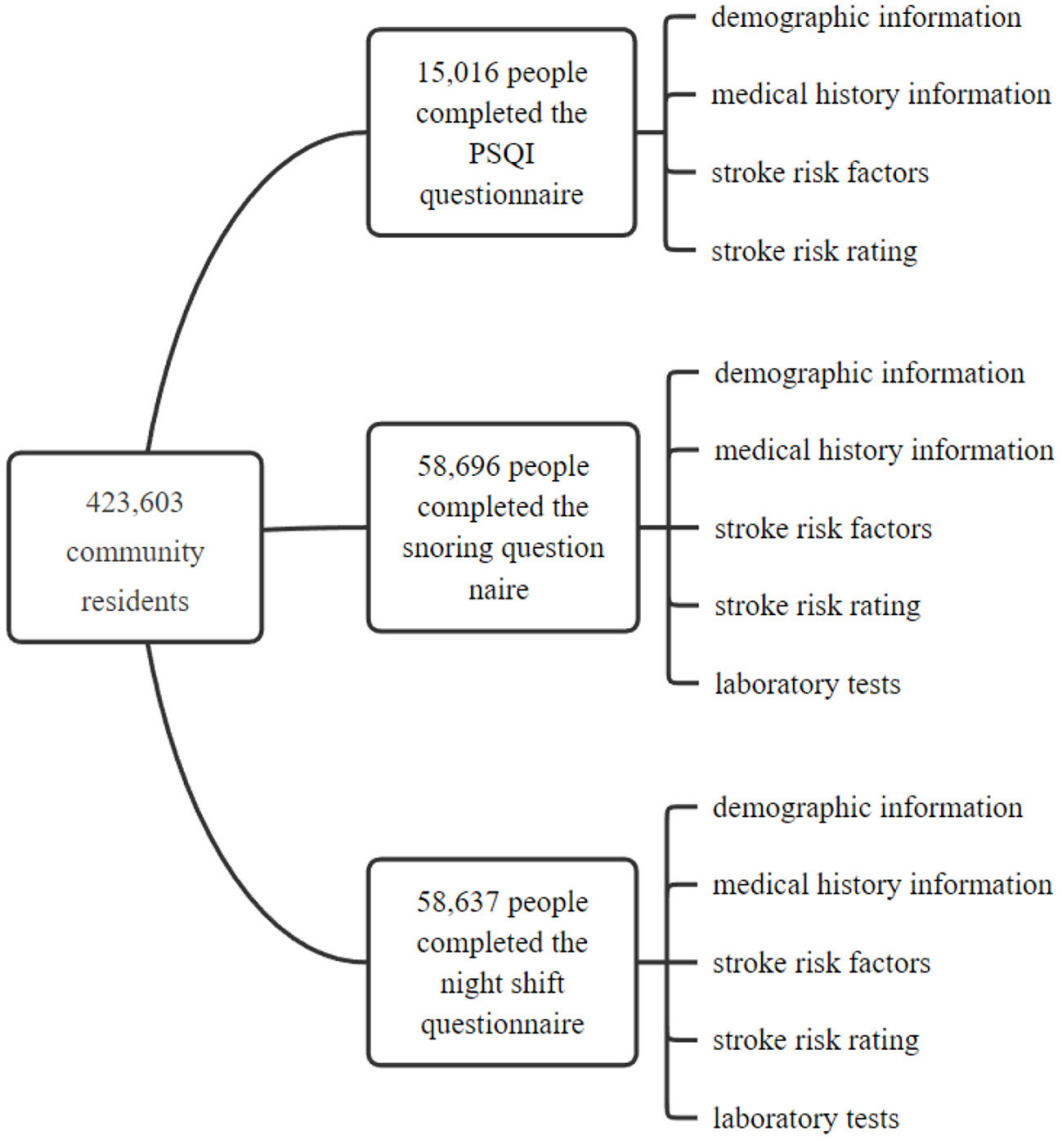
The flow chart of the research method.

Among the 423,603 community residents, 15,016 people completed the PSQI questionnaire, 58,696 people completed the snoring questionnaire, and 58,637 people completed the night shift questionnaire. In this study, demographic information, medical history information, stroke risk factors and stroke risk rating (high-risk, medium-risk and low-risk) were analyzed based on the questionnaire survey results of these three groups of people. Laboratory tests were carried out on night shifts and snorers. The flow chart of the research method was shown in Figure 1.

All participants in this study were trained strictly, and qualified physicians served as investigators or auditors. The investigators were responsible for all those who met the inclusion criteria. Auditors strictly reviewed all the input data, and checked the data according to the original files after finding extreme data, unreasonable data and deviation data. The quality control experts would conduct the quality control spot check on the data.

Sleep quality was assessed according to the PSQI questionnaire. PSQI≥5 was considered to have poor sleep quality. Snoring was defined as snoring at least 3 nights a week. Night shift was referred to regular evening or night work, lasting at least six months. Retirees were subject to their pre-retirement state.

## Statistics

In this study, descriptive analysis was used to compare the differences in demographic characteristics, lifestyle, stroke risk factors and other aspects between low and high sleep quality groups, snoring and non-snoring groups, night shift and non-night shift groups.

Enumeration data was expressed as percentage and measurement data was expressed as mean ± standard deviation ($\bar{x}\pm s$). Rates were compared using χ^2^ test. The non-parametric test method was used for the data with non-normal distribution and heterogeneity of variance. Levene test for homogeneity of variance was conducted for the comparison of the mean values between the two groups. If the variance was homogeneous, t test was used; if the variance was not homogeneous, t 'test was used. SPSS 21.0 statistical software was used for analysis and processing. *p* < 0.05 was considered statistically significant.

## Result

**Figure 2:**
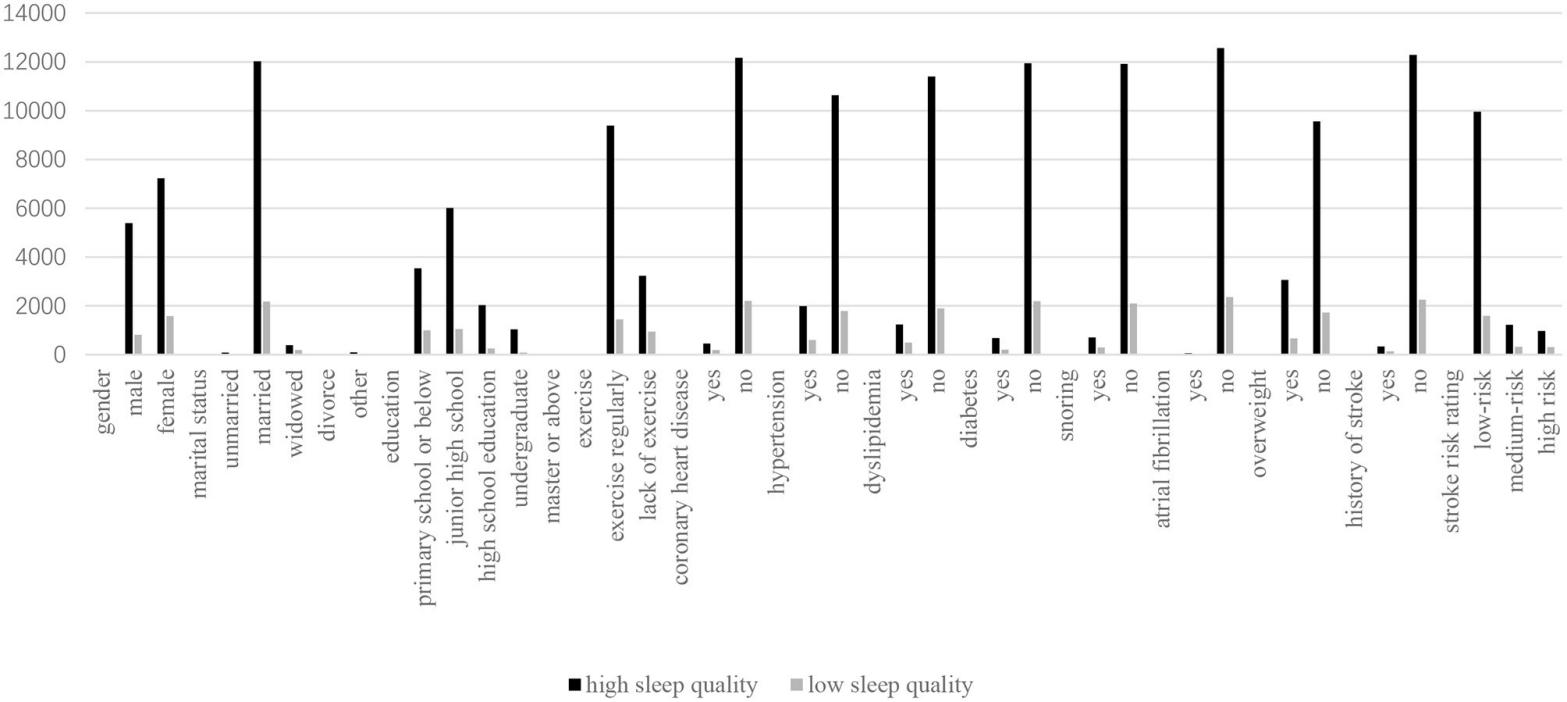
Comparison of low sleep quality and high sleep quality.

A total of 15,016 people were included in the “Sleep and Stroke Related Study” and received the PSQI questionnaire survey, among which 12,625 people (84.1%) had high sleep quality and 2,391 people (15.9%) had low sleep quality. The comparison between low and high sleep quality groups was shown in Table 1 and Figure 2. The proportion of low sleep quality was higher in women, older, widowed, people with low education (primary school or below), and people who was lack of exercise (*p* < 0.05). The analysis of stroke risk factors showed that compared with the group with high sleep quality, the proportion of coronary heart disease, hypertension, hyperlipidemia, diabetes, stroke, snoring, atrial fibrillation, overweight and stroke risk assessment as high risk were higher in the group with low sleep quality (*p* < 0.05).

**Table 1:** Comparison of low sleep quality and high sleep quality.

	**High sleep quality**	**Low sleep quality**	***p* value**
	***n*** **(%)**	***n*** **(%)**	
**Gender**			<0.001^*^
Male	5,392 (42.7)	813 (34.0)	
Female	7,233 (52.3)	1,578 (66.0)	
**Age (year)**	60.85 ± 11.01	64.18 ± 10.35	<0.001^*^
**Marital status**			<0.001^*^
Unmarried	86 (0.7)	12 (0.5)	
Married	12,022 (95.2)	2,176 (91)	
Widowed	385 (3.0)	192 (8.0)	
Divorce	34 (0.3)	7 (0.3)	
Other	97 (0.8)	4 (0.2)	
**Education**			<0.001^*^
Primary school or below	3,542 (28.1)	991 (41.4)	
Junior high school	6,009 (47.6)	1,050 (43.9)	
High school education	2,034 (16.1)	260 (10.9)	
Undergraduate	1,031 (8.2)	88 (3.7)	
Master or above	8 (0.1)	2 (0.1)	
**Drinking**			0.594
No drinking	11,458 (90.8)	2,182 (91.3)	
Heavy drinking	156 (1.2)	26 (1.1)	
Drinking habit, no heavy drinking	901 (7.1)	158 (6.6)	
Used to drink, but no longer	110(0.9)	25(1.0)	
**Exercise**			<0.001^*^
Exercise regularly	9,387 (74.4)	1,444 (60.4)	
Lack of exercise	3,238 (25.6)	947 (39.6)	
**Coronary heart disease**			<0.001^*^
Yes	457 (3.6)	190 (8.0)	
No	12,168 (96.4)	2,201 (92.0)	
**Hypertension**			<0.001^*^
Yes	1,995 (15.8)	594 (24.8)	
No	10,628 (84.2)	1,797 (75.2)	
**Dyslipidemia**			<0.001^*^
Yes	1,231 (9.8)	494 (20.7)	
No	11,394 (90.2)	1,897 (79.3)	
**Diabetes**			<0.001^*^
Yes	679 (5.4)	207 (8.7)	
No	11,946 (94.6)	2,184 (91.3)	
**Snoring**			<0.001^*^
Yes	704 (5.6)	294 (12.3)	
No	11,921 (94.4)	2,097 (87.7)	
**Atrial fibrillation**			<0.001^*^
Yes	63 (0.5)	34 (1.4)	
No	12,562 (99.5)	2,357 (98.6)	
**Smoking**			0.973
Yes	1,545 (12.2)	292 (12.2)	
No	11,080 (87.8)	2,099 (87.8)	
**Overweight**			<0.001^*^
Yes	3,063 (24.3)	670 (28.0)	
No	9,562 (75.7)	1,721 (78.0)	
**History of stroke**			<0.001^*^
Yes	333 (2.6)	141 (5.9)	
No	12,292 (97.4)	2,250 (94.1)	
**History of TIA**			0.306
Yes	148 (1.2)	34 (1.4)	
No	12,477 (98.8)	2,357 (98.6)	
**Stroke risk rating**			<0.001^*^
Low-risk	9,961 (82.0)	1,591 (71.7)	
Medium-risk	1,223 (10.0)	316 (14.2)	
High risk	969 (8.0)	312 (14.1)	

TIA, transient ischemic attack;

* p < 0.05.

**Figure 3:**
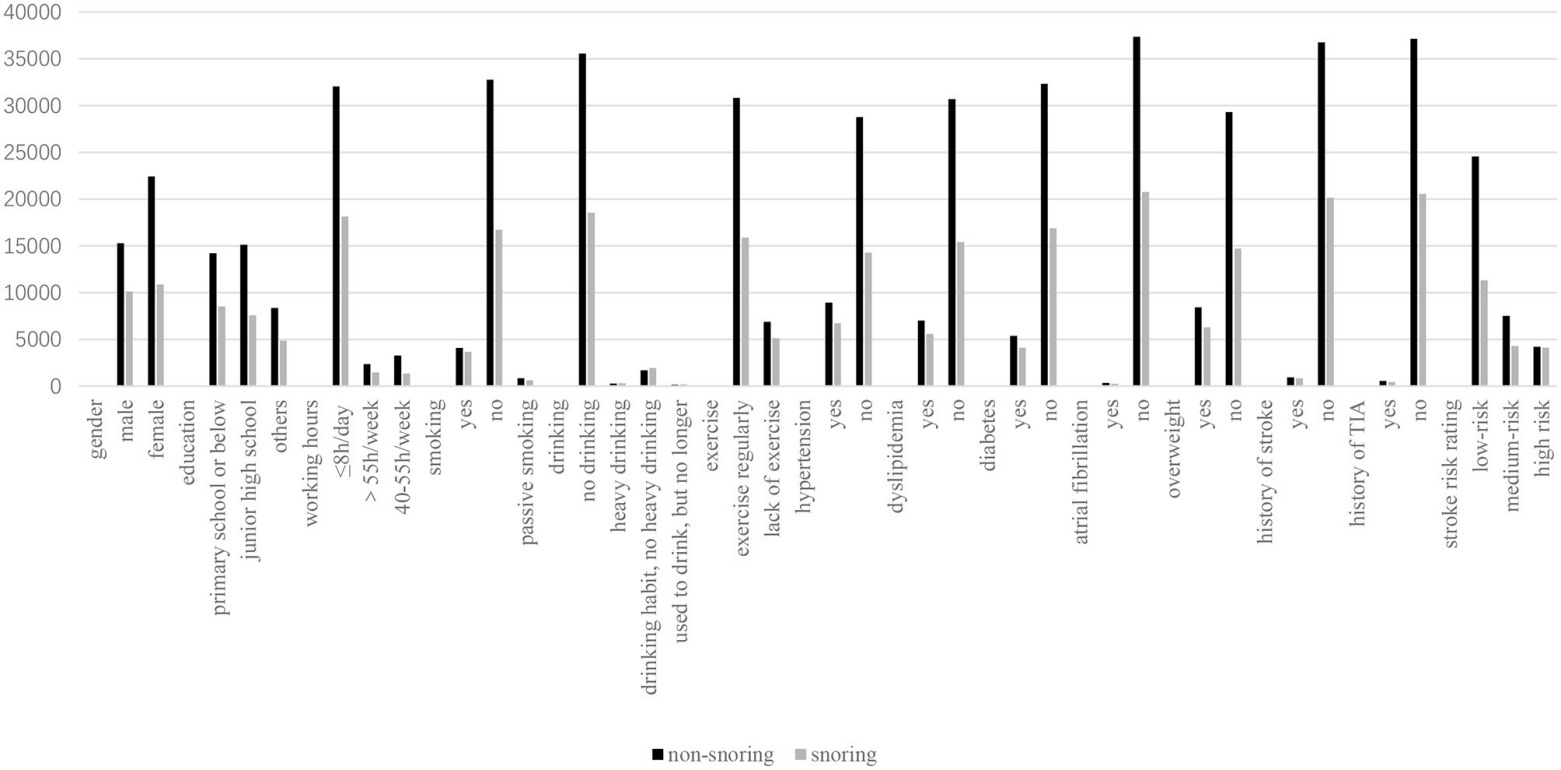
The analysis of snoring patients.

There were 58,696 people completed the snoring questionnaire. There were 37,703 non -snorers (64.2%) and 20,993 snorers (35.8%). The analysis of snorers and non-snorers was shown in Table 2 and Figure 3. Compared with non-snorers, snorers were more likely to be male, low education (primary school level and below), older, long working hours, smoking, drinking, and lack of exercise (*p* < 0.001). Compared with non-snoring group, the levels of BMI, waist circumference, neck circumference, systolic blood pressure, TG, TC, LDL and Hcy were higher in snoring group (*p* < 0.001). The levels of diastolic blood pressure, pulse, fasting blood glucose, glycated hemoglobin and HDL were lower in the snoring group (*p* < 0.05). The snoring group had higher rates of hypertension, hyperlipidemia, diabetes, atrial fibrillation, overweight, history of TIA, and stroke risk assessment (*p* ≤ 0.001).

**Table 2:** The analysis of snoring patients.

	**Non-snoring**	**Snoring**	***p***
	***n*** **(%)**	***n*** **(%)**	
**Gender**			<0.001^*^
Male	15,274 (40.5)	10,125 (48.2)	
Female	22,429 (59.5)	10,868 (51.8)	
**Age (year)**	60.36 ± 11.50	61.17 ± 11.09	<0.001^*^
**Marital status**			0.055
Unmarried	184 (0.5)	114(0.5)	
Married	36,667 (97.3)	20,451 (97.5)	
Others	821 (2.2)	399 (2.0)	
**Education**			<0.001^*^
Primary school or below	14,202 (37.7)	8,513 (40.6)	
Junior high school	15,120 (40.1)	7,575 (36.1)	
Others	8,350 (22.2)	4,876 (23.3)	
**Working hours**			<0.001^*^
≤ 8 h/day	32,059 (85.1)	18,138 (86.5)	
>55 h/week	2,361 (6.3)	1,475 (7.0)	
40–55 h/week	3,251 (8.6)	1,351 (6.5)	
**Night shift**			0.615
Yes	2,247 (6.0)	1,229 (5.9)	
No	35,424 (94.0)	19,735 (94.1)	
**Smoking**			<0.001^*^
Yes	4,087 (10.8)	3,662 (17.4)	
No	32,778 (87.0)	16,711 (79.6)	
Passive smoking	838 (2.2)	620 (3.0)	
**Drinking**			<0.001^*^
No drinking	35,559 (94.3)	18,546 (88.3)	
Heavy drinking	281 (0.7)	320 (1.5)	
Drinking habit, no heavy drinking	1,704 (4.5)	1,952 (9.3)	
Used to drink, but no longer	159 (0.5)	175 (0.9)	
**Exercise**			<0.001^*^
Exercise regularly	30,827 (81.8)	15,880 (75.6)	
Lack of exercise	6,876 (18.2)	5,113 (24.4)	
**Hypertension**			<0.001^*^
Yes	8,914 (23.6)	6,730 (32.1)	
No	28,787 (76.4)	14,261 (67.9)	
**Dyslipidemia**			<0.001^*^
Yes	6,999 (18.6)	5,593 (26.6)	
No	30,702 (81.4)	15,398 (73.4)	
**Diabetes**			<0.001
Yes	5,376 (14.3)	4,107 (19.6)	
No	32,325 (85.7)	16,884 (80.4)	
**Atrial fibrillation**			0.001^*^
Yes	337 (8.9)	248 (1.2)	
No	37,364 (99.1)	20,743 (98.8)	
**Overweight**			<0.001^*^
Yes	8,408 (22.3)	6,269 (29.9)	
No	29,292 (77.7)	14,722 (70.1)	
**History of stroke**			<0.001^*^
Yes	945 (2.5)	850 (4.0)	
No	36,756 (97.5)	20,143 (96.0)	
**History of TIA**			<0.001^*^
Yes	551 (1.5)	440 (2.1)	
No	37,150 (98.5)	20,553 (97.9)	
**Stroke risk rating**			<0.001^*^
Low-risk	24,551 (67.7)	11,324 (57.4)	
Medium-risk	7,515 (20.7)	4,317 (21.9)	
High risk	4,193 (11.6)	4,097 (20.8)	
BMI	24.35 ± 3.40	25.01 ± 3.31	<0.001^*^
Waistline (cm)	84.66 ± 9.08	86.35 ± 9.66	<0.001^*^
Neck circumference (cm)	33.77 ± 3.48	34.38 ± 3.94	<0.001^*^
Systolic pressure (mmHg)	130.45 ± 15.04	132.26 ± 16.32	<0.001^*^
Diastolic blood pressure (mmHg)	89.75 ± 9.31	81.46 ± 10.40	<0.001^*^
Pulse	74.43 ± 8.22	72.67 ± 6.63	<0.001^*^
Fasting blood-glucose (mmol/L)	5.35 ± 1.17	5.17 ± 1.37	<0.001^*^
Postprandial blood glucose at 2 h (mmol/L)	6.06 ± 1.61	6.18 ± 1.49	0.162
Glycated hemoglobin (%)	6.34 ± 2.50	6.12 ± 1.15	0.003^*^
TG (mmol/L)	1.55 ± 0.97	1.66 ± 1.07	<0.001^*^
TC (mmol/L)	4.33 ± 1.14	4.59 ± 1.15	<0.001^*^
LDL (mmol/L)	2.50 ± 0.86	2.61 ± 0.91	<0.001^*^
HDL (mmol/L)	1.50 ± 0.58	1.45 ± 0.51	<0.001^*^
Hcy (umol/L)	13.83 ± 9.86	15.47 ± 12.67	<0.001^*^

TG, triglyceride; TC, total cholesterol; LDL, low density lipoprotein; HDL, high-density lipoprotein; Hcy, homocysteine;

* p <0.05.

**Figure 4:**
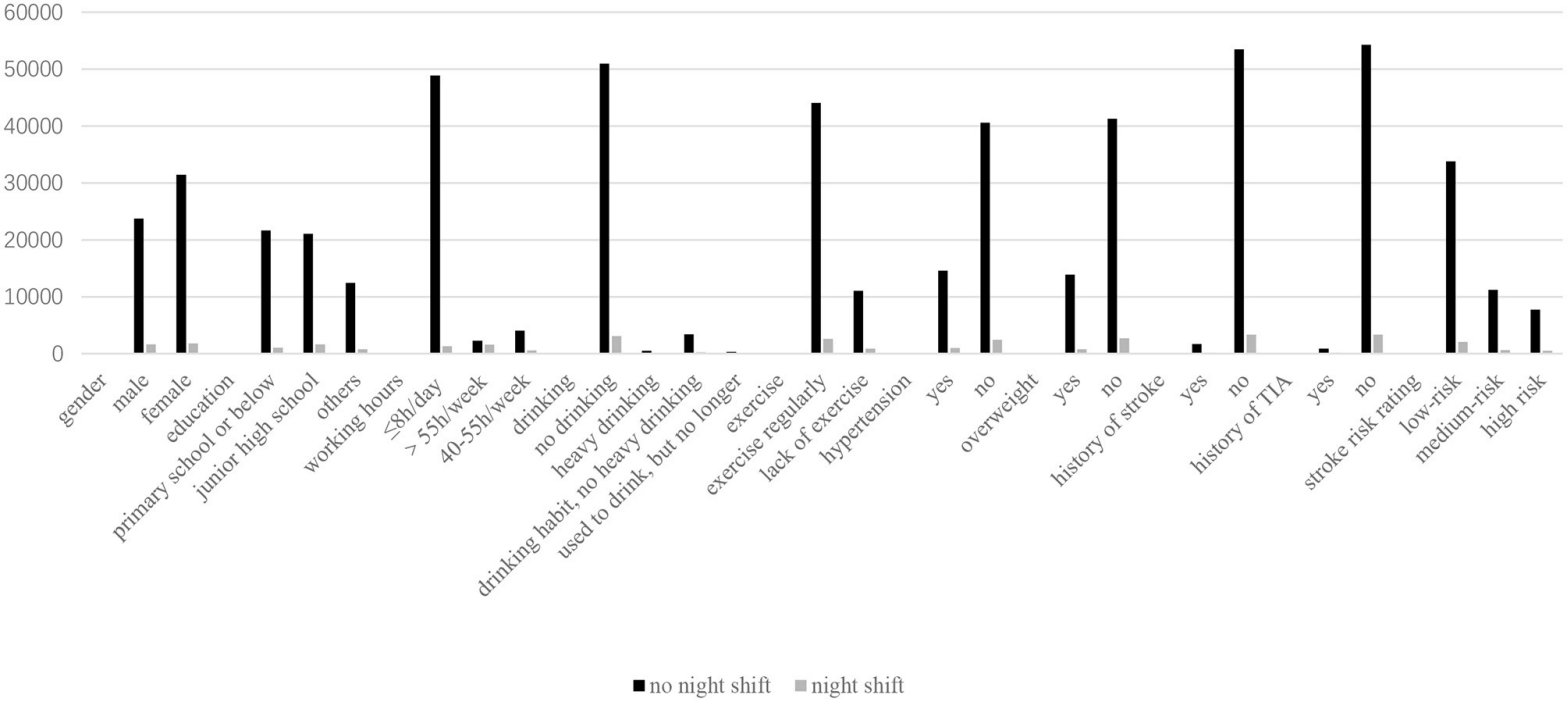
Analysis of night shift population.

There were 58,637 people completed the night shift questionnaire. There were 55,161 people (94.1%) without night shift and 3,476 people (5.9%) on the night shift. The analysis of night shift and non-night shift population was shown in Table 3 and Figure 4. Compared with the group without night shift, the proportion of male, middle school education, working more than 40 h per week and lack of exercise was higher in the group with night shift (*p* ≤ 0.001), and the proportion of primary and middle school education or less, working 8 h per day or less and no-drinking was lower in the group with night shift (*p* < 0.001). Compared with the group without night shift, the waist circumference, neck circumference and diastolic blood pressure of night shift group were higher (*p* < 0.05). The levels of BMI index, pulse, fasting blood glucose, 2 h postprandial blood glucose, glycated hemoglobin, TG, TC, LDL and Hcy in night shift group were lower, and the proportion of overweight was lower (*p* < 0.01). The night shift group had a higher risk of hypertension, history of TIA and stroke (*p* < 0.001).

**Table 3:** Analysis of night shift population.

	**No night shift**	**Night shift**	***p* value**
	***n*** **(%)**	***n*** **(%)**	
**Gender**			<0.001^*^
Male	23,719 (43)	1,657 (47.7)	
Female	31,442 (57)	1,819 (52.3)	
**Age (year)**	60.64 ± 11.37	60.62 ± 11.05	0.913
**Marital status**			0.084
Unmarried	284 (0.5)	14(0.4)	
Married	53,733 (97.4)	3,373 (97)	
Others	1,131 (2.1)	89 (2.6)	
**Education**			<0.001^*^
Primary school or below	21,659 (39.3)	1,055 (30.4)	
Junior high school	21,070 (38.2)	1,625 (46.7)	
Others	12,432 (22.5)	796 (22.9)	
**Working hours**			<0.001^*^
≤ 8 h/day	48,875 (88.6)	1,324 (38.1)	
>55 h/week	2,259 (4.1)	1,577 (45.4)	
40–55 h/week	4,027 (7.3)	575 (16.5)	
**Smoking**			0.718
Yes	7,294 (13.2)	446 (12.8)	
No	46,498 (84.3)	2,939 (84.6)	
Passive smoking	1,367 (2.5)	91 (2.6)	
**Drinking**			<0.001^*^
No drinking	50,949 (92.4)	3,096 (89.1)	
Heavy drinking	522 (0.9)	79 (2.3)	
Drinking habit, no heavy drinking	3,392 (6.1)	263 (7.6)	
Used to drink, but no longer	296 (0.5)	38 (1.1)	
**Exercise**			<0.001^*^
Exercise regularly	44,083 (79.9)	2,596 (74.7)	
Lack of exercise	11,076 (20.1)	880 (25.3)	
**Hypertension**			<0.001^*^
Yes	14,584(26.4)	1,016 (29.2)	
No	40,573 (73.6)	2,460 (70.8)	
**Dyslipidemia**			0.202
Yes	11,851 (21.5)	715 (20.6)	
No	43,306 (78.5)	2,761 (79.4)	
**Diabetes**			0.190
Yes	8,923 (16.2)	533 (15.3)	
No	46,234 (83.8)	2,943 (84.7)	
**Atrial fibrillation**			0.441
Yes	545 (1.0)	39 (1.1)	
No	54,612 (99)	3,437 (98.9)	
**Overweight**			0.001^*^
Yes	13,875 (25.2)	788 (22.7)	
No	41,282 (74.8)	2,688 (77.3)	
**History of stroke**			0.028^*^
Yes	1,666 (3.0)	128 (3.7)	
No	53,492 (97)	3,348 (96.3)	
**History of TIA**			<0.001^*^
Yes	868 (1.6)	123 (3.5)	
No	54,290 (98.4)	3,353 (96.5)	
**Stroke risk rating**			0.003^*^
Low-risk	33,787 (61.3)	2,079 (59.8)	
Medium-risk	11,195 (20.3)	626 (18)	
High risk	7,723 (14)	530 (15.2)	
BMI	24.36 ± 3.41	22.02 ± 3.32	<0.001^*^
Waistline (cm)	85.30 ± 9.33	86.35 ± 9.66	0.015^*^
Neck circumference (cm)	32.94 ± 3.70	34.77 ± 3.13	<0.001^*^
Systolic pressure (mmHg)	131.07 ± 15.56	131.396 ± 15.12	0.224
Diastolic blood pressure (mmHg)	80.25 ± 9.72	82.01 ± 10.09	<0.001^*^
Pulse	74.43 ± 8.22	72.67 ± 6.63	<0.001^*^
Fasting blood-glucose (mmol/L)	5.35 ± 1.18	5.18 ± 1.38	<0.001^*^
Postprandial blood glucose at 2h (mmol/L)	6.27 ± 1.59	5.54 ± 1.29	<0.001^*^
Glycated hemoglobin (%)	6.28 ± 2.31	6.00 ± 0.94	<0.001^*^
TG (mmol/L)	1.60 ± 1.00	1.42 ± 1.14	<0.001^*^
TC (mmol/L)	4.45 ± 1.15	4.03 ± 1.17	<0.001^*^
LDL (mmol/L)	2.56 ± 0.86	2.27 ± 1.10	<0.001^*^
HDL (mmol/L)	1.49 ± 0.56	1.30 ± 0.44	<0.001^*^
Hcy (umol/L)	14.68 ± 11.44	12.61 ± 6.79	<0.001^*^

TG, triglyceride; TC, total cholesterol; LDL, low density lipoprotein; HDL, high-density lipoprotein; Hcy, homocysteine;

* p <0.05.

## Discussion

In this study, we used the PSQI questionnaire, which is recognized as a reliable, effective and standardized global sleep quality measurement questionnaire and have previously been confirmed for its accuracy. PSQI was developed in 1989 by Dr. Buysse, a psychiatrist at the University of Pittsburgh. It was used to evaluate sleep quality in clinical and basic research. Liu Xianchen translated the scale into Chinese in 1996 and conducted a study on its reliability and validity. The results showed that the scale also had high reliability and validity when applied to China. The PSQI is a self-reported questionnaire used to assess sleep quality. It contains 19 questions which indicate overall sleep quality. Each question weighed on a 0–3 interval scale in 7 components. PSQI < 5 indicates high sleep quality, and PSQI ≥5 indicates low sleep quality. In this study, 15,016 people were surveyed by PSQI questionnaire, and 2,391 people had poor sleep quality, accounting for 15.9% of the community population over 40 years old.

Individuals who lack support from spouses and families may have difficulty controlling their anxiety and loneliness, causing sleep disorders. Consistent with this, sleep quality was lower in the widowed population in our study. Some studies had shown that the sleep quality of female population is lower (Da Rocha et al., 2013). This study showed that the lower sleep quality of female patients was related to the more trivial life and the lower stress resistance of female patients. In addition, consistent with previous studies, our study also found that people with lower education level had lower sleep quality, which was considered to be related to cognitive level, life stress, health literacy, stress resistance, and psychological factors. Physical activity may affect sleep quality in adults (Kredlow et al., 2016). Regular non-competitive physical activities can lower body temperature, increase parasympathetic activity, down-regulate hypothalamic-pituitary-adrenal secretion, increase melatonin secretion, regulate the release of inflammatory cytokines, reduce psychological stress, and improve sleep quality (Chennaoui et al., 2015). Our study showed that people who were physically inactive had higher rates of poor sleep quality. Therefore, people with poor sleep quality can improve their sleep with aerobic exercise or intensive exercise. Large waist circumference and neck circumference may cause sleep-disordered breathing (Ng et al., 2015; Baltzis et al., 2016). Our research showed that people who were overweight had lower sleep quality.

Our study shows that people with hypertension have lower sleep quality. A study on hypertension in rural adults in northeast China (Liu et al., 2016) found that the prevalence of hypertension was significantly increased in people with low sleep quality. However, some studies have reported that the PSQI score was not correlated with hypertension (Sforza et al., 2014). Decreased sleep quality is an important risk factor for hypertension, possibly due to increased sympathetic nervous system activity.

Sleep disorders have been reported to be associated with elevated glycated hemoglobin in type 2 diabetes (Knutson et al., 2006). A meta-analysis showed that the risk of diabetes due to sleep deprivation increased with the extension of follow-up time (Cappuccio et al., 2010). However, an earlier study of 6,509 Japanese workers aged 19–69 found no significant association between sleep deprivation and the development of diabetes (Hayashino et al., 2007). Our study shows that people with diabetes have lower sleep quality. Sleep deprivation leads to the increase of various inflammatory markers and the decrease of immune level, thus promoting the occurrence and development of diabetes (Wang et al., 2013).

The relationship between sleep quality and lipid levels remains controversial. Studies have shown that lipid levels are only related to sleep duration, not self-reported sleep quality (Petrov et al., 2013). However, some studies have shown that hyperlipidemia is independently associated with decreased subjective sleep quality (Cappuccio et al., 2010). Studies have shown that higher LDL and TG levels are also associated with higher PSQI scores (Geovanini et al., 2019). However, some studies showed no significant correlation between PSQI score and blood lipid (Petrov et al., 2013). Our study showed that people with poor sleep quality have higher rates of hyperlipidemia. Decreased sleep quality leads to increased activity of hypothalamic-pituitary-adrenal axis and increased cortisol secretion, resulting in increased cholesterol level (Zhan et al., 2014).

Studies have found that decreased sleep quality and shorter sleep duration are independently associated with the risk of coronary heart disease events in adults 40 years and older (Lao et al., 2018). Everding et al. (2016) used PSQI to evaluate sleep quality and found a significant correlation between sleep quality and risk factors of cardiovascular disease. Our study also showed that poor sleep quality was associated with coronary heart disease. The mechanisms by which sleep disorders affect cardiovascular disease include: first, sleep deprivation leads to changes in leptin and ghrelin levels, which promote the development of obesity, and elevated blood sugar levels; second, sleep disorders can lead to changes in growth hormone metabolism and increased cortisol secretion; finally, mild inflammation caused by sleep disorders can increase the hypothalamic-pituitary-adrenal axis stress response, resulting in increased blood pressure, blood flow blockage, and increased risk of cardiovascular disease (Klop et al., 2013).

Stroke patients are prone to sleep disorders and may relapse several years after stroke (Jönsson et al., 2006). One research found that participants who slept for <6 h had a 0.97 times increased risk of stroke compared to those who slept for 6–7 h and participants with sleep disorders had 0.71 times the risk of stroke compared with those without sleep disorders (Wang and Ren, 2022). Our study found that people with a history of stroke and who were assessed to be at high risk of stroke had lower sleep quality. Sleep disorders are associated with a variety of chronic diseases, including hypertension, diabetes, dyslipidemia and so on, all of which are the causes of stroke. Sleep and stroke also affect each other.

The majority of snoring people are male, and this sex difference may be related to the difference of pharynx collapse and central respiratory drive. The incidence of snoring in males was higher than that in females. In addition, habitual snoring is associated with high BMI (Svensson et al., 2006). In this study, the BMI, waist circumference and neck circumference of snoring people were higher than the none snoring people. Our study also found that people with low education (primary school level or below) and working more than 55 h per week were more likely to snore. These factors and high psychological stress could indirectly lead to higher rates of smoking and drinking.

This study showed that people with hypertension, hyperlipidemia, coronary heart disease, atrial fibrillation and diabetes had a high proportion of snoring. The fasting blood glucose, TG, TC, LDL and Hcy of snoring people were higher than those of non-snoring people, and the HDL level of snoring people was lower than that of non-snoring people. Snoring has been linked to hypertension, type 2 diabetes and metabolic syndrome, all of which can increase the risk of coronary heart disease and stroke. Severe snoring is associated with increased plaque and atherosclerosis (Drager and Lorenzi-Filho, 2008). This study showed that the proportion of snoring was higher in TIA or the high-risk group of stroke. A study showed that habitual snoring was associated with a 26% increased risk of stroke and a 15% increased risk of coronary heart disease (Li et al., 2014). Habitual snoring has been identified as an independent risk factor for stroke (Partinen, 1995). This study showed that the snoring group had lower fasting blood glucose and glycated hemoglobin, but a higher incidence of diabetes, possibly because the diabetic patients had normal levels of fasting blood glucose and glycated hemoglobin under the control of medication.

Sleep insufficiency is a common problem in the night shift population. Our study showed that the proportion of working <8 h and sleeping more than 8 h in night shift population was significantly smaller than that in non-night shift population. Sleep insufficiency can adversely affect carbohydrate metabolism and levels of endocrine hormones such as insulin, cortisol and leptin, which can lead to changes in appetite and glucose metabolism, accelerating the development of obesity and diabetes. Sleep restriction in adult males who sleep normally leads to increased sympathetic, norepinephrine, and pro-inflammatory cytokines (interleukin-1, interleukin-6, and C-reactive protein) activity, which are independently associated with coronary heart disease and death (Dettoni et al., 2012).

Night shift work disrupts circadian rhythm and is associated with increased risk factors for vascular disease and catecholamine secretion (Costa et al., 1997). The association between night shift work and cerebrovascular disease has not been fully established. A report from the Nurses' Health Study showed that night nurses had a 4% increased risk of ischemic stroke (Brown et al., 2009). Studies have also pointed out that people who work at night have a higher risk of dyslipidemia, metabolic syndrome, hypertension and diabetes, and even one overnight shift can lead to increased blood pressure and impair heart rate variability (Lo et al., 2010).

A characteristic of night shift work is its susceptibility to metabolic syndrome (Sookoian et al., 2007). Some studies have shown that night shift workers have an elevated BMI, however, some studies have shown a decrease in BMI after a short time of night shift while no change in BMI after a long time of night shift (van Amelsvoort et al., 2004). These studies show that adaptability increases after long time of night shifts. Our study showed that BMI index, pulse, fasting blood glucose, 2 h postprandial blood glucose, glycosylated hemoglobin, TG, TC, LDL, HDL and Hcy levels of night shift population were lower than those of non-night shift population, which was different from the results of previous studies showing that night shift population was prone to metabolic disorders. The reason may be that the subjects of this study are the people who have worked at night for more than half a year, and their adaptability is enhanced after working at night for a long time. Secondly, some of the subjects have retired and are not disturbed by night work, so their metabolic status tends to be stable. Further prospective studies are needed to confirm this.

## Conclusion

1. Women, the widowed, low education, and the elderly are more likely to have poor sleep quality. Compared with high sleep quality, low sleep quality may be associated with hypertension, hyperlipidemia, diabetes, snoring, coronary heart disease, atrial fibrillation and other risk factors of stroke.
2. The Chinese community over 40 years old have a higher proportion of snoring. A higher proportion of risk factors of stroke and lower sleep quality were found in snore population. Snoring may be related to the risk factors of stroke.
3. Night shift may be associated with risk factors of stroke. After a long time of night shift, the adaptability increased, and the metabolic situation tended to be stable. The levels of BMI, pulse, fasting blood glucose, 2 h postprandial blood glucose, glycosylated hemoglobin, TG, TC, LDL, HDL and Hcy in the night shift population were lower than those in the non-night shift population.

## Limitation

1. Sleep quality and snoring were obtained by questionnaire, and there was no objective measurement, which could produce potential bias.
2. This is a cross-sectional study and cannot determine a definitive cause-and-effect relationship.

## Data availability statement

The original contributions presented in the study are included in the article/supplementary material, further inquiries can be directed to the corresponding author.

## Ethics statement

The ethical approval and consent was obtained from the Ethics Committee of Tianjin Huanhu Hospital. All methods were carried out in accordance with relevant guidelines and regulations. All experimental protocols were approved by Tianjin Huanhu Hospital Licensing Committee. Informed consent was obtained from all subjects.

## Author contributions

YZ was responsible for writing the article. TZ, XX, YH, CZ, RL, YY, XL, and WY are responsible for collecting and organizing data. WY was responsible for reviewing the articles. All authors contributed to the article and approved the submitted version.
